# Metamemory for pictures of naturalistic scenes: Assessment of accuracy and cue utilization

**DOI:** 10.3758/s13421-021-01170-5

**Published:** 2021-04-02

**Authors:** Monika Undorf, Arndt Bröder

**Affiliations:** Department of Psychology, School of Social Sciences, University of Mannheim, A 5, 6, C 203, 68159 Mannheim, Germany

**Keywords:** Metamemory, Judgments of learning, Pictures, Naturalistic scenes, Cue integration

## Abstract

Memory for naturalistic pictures is exceptionally good. However, little is known about people’s ability to monitor the memorability of naturalistic pictures. We report the first systematic investigation into the accuracy and basis of metamemory in this domain. People studied pictures of naturalistic scenes, predicted their chances of recognizing each picture at a later test (judgment of learning, JOL), and completed a recognition memory test. Across three experiments, JOLs revealed substantial accuracy. This was due to people basing their JOLs on multiple cues, most of which predicted recognition memory. Identified cues include intrinsic picture attributes (e.g., peacefulness of scenes; scenes with or without persons) and extrinsic aspects of the study situation (e.g., presentation frequency; semantic distinctiveness of scenes with respect to the context). This work provides a better understanding of metamemory for pictures and it demonstrates close parallels between metamemory for naturalistic scenes and verbal materials.

Much of memory’s power is due to the skillful ways in which people strategically regulate their memories based on *metamemory—*assessments of their own learning and remembering (Benjamin, 2007). There is much evidence that metamemory plays a crucial role in successful learning and remembering of verbal materials (see, e.g., Dunlosky & Metcalfe, 2009; Koriat, 2007; Undorf et al., 2020). In contrast, relatively little is known about metamemory for other materials and, in particular, pictures of naturalistic scenes (e.g., Rhodes, 2016). This lack of research is surprising because memory for naturalistic pictures is exceptionally good (e.g., Nickerson, 1965; Shepard, 1967; Standing, 1973). Investigating whether or not it is complemented by accurate metamemory is therefore an intriguing research question. The current research aims to expand our knowledge about metamemory by investigating the accuracy and basis of people’s predictions of remembering recently studied pictures of naturalistic scenes at a later test (judgments of learning [JOLs]). Thus, this research addresses the generalizability of established principles and findings in the literature on metamemory to stimuli researchers have rarely studied.

For verbal materials, immediate JOLs are moderately accurate (e.g., Bröder & Undorf, 2019; Dunlosky & Metcalfe, 2009; Koriat, 1997).^1^ This accuracy is due to people basing their JOLs on probabilistic cues, many of which are predictive of actual memory (Koriat, 1997; Rhodes, 2016; Undorf et al., 2018; Undorf & Bröder, 2020). The cues that underlie JOLs are often classified into different groups (Koriat, 1997). *Intrinsic cues* are inherent to and inseparable from the study items; examples include word concreteness or word frequency. *Extrinsic cues* are bound to specific study conditions and can be randomly assigned to study items; examples include presentation time or the frequency of study presentations. Evidence is accumulating that people base their JOLs for verbal materials not only on single cues but on multiple intrinsic and extrinsic cues simultaneously (e.g., Bröder & Undorf, 2019; Tatz & Peynircioğlu, 2019; Undorf et al., 2018). For instance, Undorf et al.’s (2018) participants studied single words and made JOLs for recalling each word at a later test. When varying the two extrinsic cues number of study presentations (1 vs. 2) and font size (18 point vs. 48 point), individual-level analyses revealed that the majority of participants based their JOLs on both cues. Similarly, people integrated the two intrinsic cues concreteness (abstract vs. concrete) and emotionality (neutral vs. emotional) in their JOLs (also see Bröder & Undorf, 2019). Further experiments showed that all four cues affected JOLs when manipulated simultaneously and confirmed that people integrated three cues that varied on a continuum in their JOLs. In summary, it is established that JOLs for verbal materials are moderately accurate and a growing number of studies indicate that JOLs for verbal materials rely on multiple intrinsic and extrinsic cues.

For pictures of naturalistic scenes, however, the accuracy and basis of JOLs has remained largely unexplored. In a study by Kao et al. (2005), people studied scenes and indicated whether they would remember or forget each scene on a later recognition test. Results revealed that JOLs accurately predicted differences in the relative memorability of scenes. Using pictures from the IAPS database (Lang et al., 2008), some of which presumably depicted scenes, Hourihan and Bursey (2017) and Tauber et al. (2017) found above-chance JOL accuracy and higher JOLs for positively valenced pictures than for neutral pictures. Schmoeger et al. (2020) complemented this finding by reporting higher JOLs for negatively valenced than for neutral IAPS pictures. Together, these findings indicate that people at least partly based their JOLs for pictures on emotional valence.

Recall memory (Schmoeger et al., 2020; Tauber et al., 2017) but not recognition memory (Hourihan & Bursey, 2017) was better for positive and negative pictures than for neutral pictures, meaning that people accurately predicted valence effects on recall but not on recognition memory (for similar findings with verbal materials, see Bröder & Undorf, 2019; Zimmerman & Kelley, 2010).

The few studies on JOLs for naturalistic pictures thus provide preliminary evidence that these judgments may be accurate and rely on probabilistic cues. Of course, the specific cues that underlie JOLs for naturalistic pictures may differ from those identified with verbal materials. Some of the cues found to underlie JOLs for words are by definition specific to verbal materials (e.g., word concreteness, word frequency, font size of study words). In contrast, other cues that form the basis of JOLs for verbal materials could, in principle, also underlie JOLs for naturalistic pictures (e.g., valence, arousal, or presentation frequency). Also, there are probably cues that are specific to JOLs for naturalistic pictures. One potential cue is whether scenes are presented in color or in grayscale (for effects of this cue on recognition memory, see Spence et al., 2006; Wichmann et al., 2002).

The research reported here aims to contribute to a better understanding of the accuracy and basis of metamemory for naturalistic pictures. Across three experiments, we simultaneously manipulated two or more cues that were expected to affect recognition memory. These expectations were based on a massive research effort on the memorability of more than 2,000 scene photographs (Bylinskii et al., 2015; Isola et al., 2011, 2014). In this research, participants completed an online continuous recognition task in which they viewed long streams of scene photographs and pressed a key whenever they recognized a scene they had seen before. Results from more than 600 participants showed that scenes systematically differed in memorability. Also, results showed that numerous picture attributes predicted memorability. For instance, scenes that independent raters judged to tell a story were more memorable than scenes that did not tell a story (Isola et al., 2011). Also, judged peacefulness of scenes affected memorability, with scenes judged as peaceful being less memorable than scenes judged as not peaceful (Isola et al., 2011). Semantic distinctiveness of scenes with respect to the context boosted memorability: Scenes were more memorable when presented in the context of scenes from other categories than in the context of scenes from the same category (e.g., memorability of a highway scene was higher when presented among scenes from 21 different categories than among highway scenes only; Bylinskii et al., 2015). Also relevant for present purposes, Isola et al. (2011, 2014) reported that it was not trivial for people to accurately assess scene memorability. Isola et al. (2014) had participants from an online pool indicate for a set of scenes whether each was or was not memorable or whether they would or would not, when seeing each scene again later, realize having seen it before. People’s binary ratings did not correlate with actual memorability (Isola et al., 2014). Also, ratings revealed that people thought aesthetic beauty and interestingness to increase memorability, whereas both picture attributes rather impaired memorability. Similar misconceptions prevailed among experts on human and computer vision (Isola et al., 2011). Notably, these general memorability ratings are so different from standard JOLs—made in a learning phase and on scales that allow learners to differentiate finely graded levels of confidence—that the accuracy of JOLs for naturalistic pictures is still an open question. Based on the literature on JOLs for verbal materials, we expected that JOLs for naturalistic pictures would show at least some accuracy. We will return to the issue of differences between Isola et al.’s (2014) memorability judgments and standard JOLs in the General Discussion.

We manipulated two extrinsic cues in Experiment 1, two intrinsic cues in Experiment 2, and a total of five cues (two extrinsic, three intrinsic) in Experiment 3. In each experiment, participants studied around 100 scene pictures, made an immediate JOL for each scene, and completed a recognition test where studied scenes were randomly intermixed with an equal number of new scenes. We examined whether and how each manipulated cue affected JOLs and investigated the relative accuracy of JOLs (e.g., Dunlosky & Metcalfe, 2009; Koriat, 2007; Rhodes, 2016). Relative accuracy (also referred to as resolution) assesses the extent to which JOLs distinguish between items people will later recognize and those they will not recognize.

## Experiment 1

In Experiment 1, we examined the accuracy and basis of JOLs for pictures of naturalistic scenes that differed in contextual semantic distinctiveness and color. To manipulate contextual distinctiveness, we varied the number of scenes that came from specific semantic categories: Distinct scenes came from categories that contributed four scenes to the study list, and indistinct scenes came from categories that contributed 28 scenes to the study list. This manipulation affected primary distinctiveness according to Schmidt (1991), because it made distinctive items stand out from the other study items (e.g., a kitchen scene stands out more than an airplane scene when four scenes depict kitchens and 28 scenes depict airplanes). High contextual distinctiveness was previously found to improve memory performance for scenes (e.g., Bylinskii et al., 2015) and to increase memory and metamemory judgments for words and face–name associations (Dunlosky et al., 2000; Watier & Collin, 2012). To manipulate color, participants studied the colored originals of half the scenes and grayscale versions of the remaining scenes.^2^ Studies show that colored scenes are remembered better than grayscale scenes (e.g., Spence et al., 2006; Wichmann et al., 2002), and a single study found that young children expect to remember sets of colored pictures of common objects better than sets of black-and-white pictures (Kreutzer et al., 1975). We therefore predicted that contextual distinctiveness and color would improve recognition memory and probably also increase JOLs.

### Method

#### Participants

We aimed at a sample size of 54 participants to obtain a statistical power of (1 − β) = .95 to detect medium-sized main effects (*f* = .25, equivalent to η_p_^2^ = .06) with α = .05 when assuming a correlation of .50 between repeated measures (all power analyses conducted via G*Power 3; Faul et al., 2007). We aimed at optimizing power for main effects because we had no hypotheses about interactions. Participants were 56 University of Mannheim undergraduates (43 females, 13 males) with a mean age of 23.05 years (*SD* = 4.41).

#### Design and materials

**Fig. 1:**
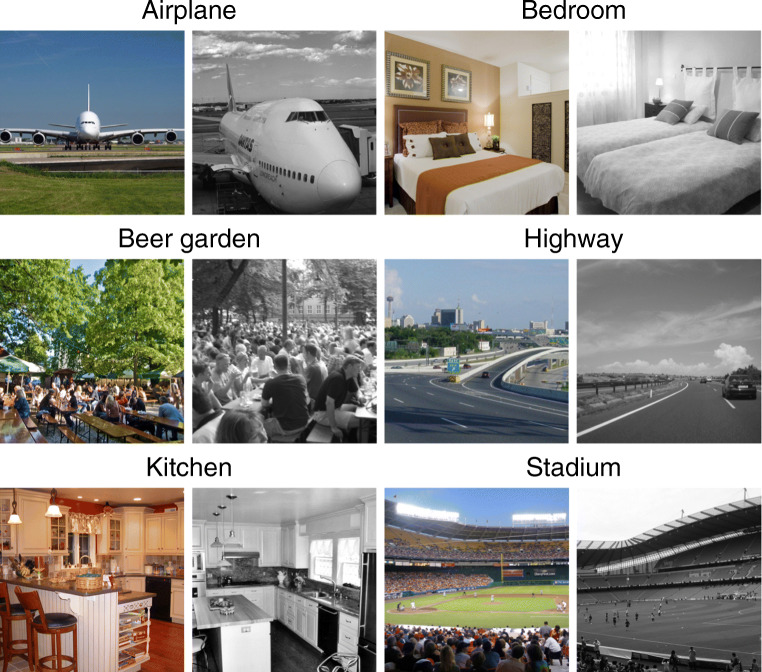
Example pictures from each of the six scene categories used in Experiment 1

The design was a 2 (distinctiveness: low vs. high) × 2 (color: grayscale vs. color) within-participants design. Stimuli were 336 pictures from the SUN database (Xiao et al., 2010), with 56 pictures from each of six semantic scene categories (airplane, bedroom, beer garden, highway, kitchen, stadium; see Fig. 1). Categories varied in whether they included indoor or outdoor scenes (indoor: kitchen and bedroom; outdoor: airplane, beer garden, highway, stadium) and whether scenes did or did not contain persons (with persons: beer garden, stadium; without persons: airplane, bedroom, highway, kitchen).

Appendix Table 3 illustrates how stimuli were distributed across within-subjects conditions. In indistinct categories, all 56 pictures served as stimuli. These pictures were divided into two parallel sets, with 28 items each. In distinct categories, a fixed set of eight randomly selected pictures served as stimuli, divided into two parallel sets with four items. For each participant, one set of scenes per category served as targets (items presented at study and test) and the second set of scenes served as distractors (new items presented at test but not at study). Scene categories and picture sets were counterbalanced across participants such that each scene category was equally often distinct and indistinct and that each picture served equally often as target and distractor. 

We created a grayscale version of each picture that was used in the grayscale condition, whereas the colored original was used in the color condition.

#### Procedure

Participants were tested individually or in small groups in the laboratory. The experiment was fully computerized and took about 30 min to complete. It consisted of a study phase and a recognition memory test. Participants studied 96 scene photographs, with four scenes from each of the three distinct categories and 28 scenes from each of the three indistinct categories. They were tested on 192 scenes that included four studied and four new scenes from each distinct category and 28 studied and 28 new scenes from each indistinct category. At study and test, participants saw one randomly selected half of the scenes per category in color and the other half in grayscale. For each participant, scenes were presented in a new random order at study and test. At study, each scene was preceded by a fixation cross (500 ms) and displayed for 1 s. Immediately afterwards, participants made a JOL by indicating the probability of recognizing the picture at test, using one of 11 keys labeled 0, 10 … 90, 100. Following the study phase, participants performed a numerical filler task for 3 min to avoid recency effects. On filler trials, participants added up 10 digits (e.g., 4234657984) and typed in the sum’s last digit (e.g., 2). This filler task was chosen, because it does not interfere with episodic memory or pictorial encoding. Participants then completed a self-paced recognition test, in which one scene after the other was presented, and participants indicated whether it was old or new by pressing labeled keys.

### Results

Contextual distinctiveness and color affected both JOLs and recognition memory (see Table 1). A 2 (distinctiveness: low vs. high) × 2 (color: grayscale vs. color) repeated-measures ANOVA on JOLs revealed a main effect of distinctiveness, *F*(1, 55) = 22.10, *p* < .001, η_p_^2^ = 0.29, indicating higher JOLs for scenes from distinct than indistinct categories as well as a main effect of color, *F*(1, 55) = 43.78, *p* < .001, η_p_^2^ = 0.44, indicating higher JOLs for color than grayscale scenes, but no interaction, *F*(1, 55) = 1.95, *p* = .168, η_p_^2^ = 0.03. A similar ANOVA on corrected hit rates *P*_r_ measuring actual recognition performance revealed main effects of distinctiveness, *F*(1, 55) = 60.00, *p* < .001, η_p_^2^ = 0.52, and color, *F*(1, 55) = 29.64, *p* < .001, η_p_^2^ = 0.35, but no interaction, *F* < 1.

**Table 1:** Descriptive statistics for Experiments 1 and 2

		JOL	% Hits	% False alarms	*P*_r_
Experiment 1					
Indistinct	Grayscale	37.04 (13.18)	56.21 (17.23)	26.57 (14.97)	.30 (.16)
	Color	42.64 (12.75)	63.05 (14.83)	23.21 (12.69)	.40 (.16)
Distinct	Grayscale	42.62 (16.19)	61.31 (23.81)	13.69 (15.28)	.48 (.27)
	Color	50.57 (16.37)	70.83 (24.46)	10.42 (14.76)	.60 (.28)
Experiment 2					
Story low	Peacefulness low	41.49 (12.36)	77.16 (14.66)	13.33 (13.25)	.64 (.21)
	Peacefulness high	42.06 (13.86)	65.99 (15.53)	15.37 (14.79)	.51 (.23)
Story high	Peacefulness low	48.82 (14.90)	82.28 (11.45)	9.01 (10.63)	.73 (.15)
	Peacefulness high	46.22 (14.52)	70.49 (15.48)	15.31 (13.67)	.55 (.21)

*JOL* judgment of learning (on a 0–100 scale); % *Hits* percentage of hits; % *False alarms* percentage of false alarms; *P*_r_ = corrected hit rate according to Snodgrass and Corwin (1988), which is computed as *p*(hit) − *p*(false alarm)

Even though contextual distinctiveness and color affected JOLs at the aggregate level, it was still possible that no participant integrated the two cues in his or her JOLs (cf. Undorf et al., 2018). The reason is that main effects of the two cues at the aggregate level could result if each participant’s JOLs were based on only one cue, but different participants based their JOLs on different cues. Two supplementary analyses therefore evaluated cue integration at the individual level. The first analysis focused on simple mean differences. A participant was scored to base JOLs on distinctiveness if his or her mean JOLs were higher for scenes from distinct than indistinct categories and was scored to base JOLs on color if his or her mean JOLs were higher for color than grayscale scenes. According to this criterion, 66% of participants integrated distinctiveness and color in their JOLs (see Table 2).

**Table 2:** Number of experimentally manipulated cues used by percentages (and absolute numbers) of individual participants in Experiments 1 to 3

	Number of cues						Cue integration
	0	1	2	3	4	5	
Experiment 1							
Mean differences	5.36 (3)	28.57 (16)	66.07 (37)				66.07 (37)
Cohen’s |*d*| ≥ .2	8.93 (5)	48.21 (27)	42.86 (24)				42.86 (24)
Experiment 2							
Mean differences	7.41 (4)	46.30 (25)	46.30 (25)				46.30 (25)
Cohen’s |*d*| ≥ .2	16.67 (9)	42.59 (23)	40.74 (22)				40.74 (22)
Experiment 3							
Mean differences	0.00 (0)	3.85 (2)	30.77 (16)	46.15 (24)	17.31 (9)	1.92 (1)	96.15 (50)
Cohen’s |*d*| ≥ .2	1.92 (1)	3.85 (2)	26.92 (14)	32.69 (17)	30.77 (16)	3.85 (2)	94.23 (49)

Mean differences = percentage of participants who based their JOLs on the given number of manipulated cues as indicated by higher JOLs for the cue level expected to yield higher JOLs; Cohen’s |*d*| ≥ .2 = percentage of participants who based their JOLs on the given number of manipulated cues as indicated by individual Cohen’s |*d*| ≥ .2 for the cue effect on JOLs

**Fig. 2:**
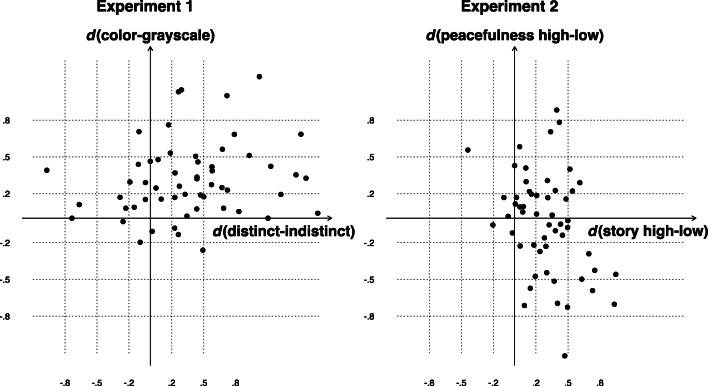
Scatterplots of individual effect sizes (Cohen’s *d*) on JOLs in Experiments 1 and 2

The second individual-level analysis was based on effect sizes for the very same mean differences. Figure 2 shows each participant’s Cohen’s *d* for distinctiveness (*x-*axis) and color (*y*-axis). The majority of participants are located in the upper right quadrant, indicating that they predicted better memory for distinct than indistinct scenes and better memory for color than grayscale scenes. When using Cohen’s (1977) small effects convention of |*d*| ≥ .2 as evidence for reliable cue effects, 43% of participants integrated the two cues in their JOLs.

Finally, we examined the relative accuracy of JOLs. The most commonly used measure of relative JOL accuracy is the within-subject gamma correlation between JOLs and memory performance (Nelson, 1984). Recently, however, using the gamma coefficient as a measure of relative accuracy has been criticized for conflating people’s metacognitive competency with random noise (Bröder & Undorf, 2019) and for inflated Type 1 errors (Murayama et al., 2014), among others (cf. Benjamin & Diaz, 2008; Masson & Rotello, 2009; Spellman et al., 2008). We therefore analyzed relative accuracy not only using the gamma correlation between JOLs and recognition hits but also using the matching index *G* from Brunswik’s (1952) lens model (Bröder & Undorf, 2019) and using a mixed-effects model analysis (Murayama et al., 2014).

A significantly positive gamma coefficient, *M* = .28 (*SD* = .21), *t*(55) = 9.83, *p* < .001, *d* = 1.33, and a significantly positive matching index *G*, *M* = .40 (*SD* = .09), *t*(55) = 4.63, *p* < .001, *d* = 0.62, indicated that JOLs captured differences in the relative memorability of scenes. The matching index *G* was numerically but not significantly higher than the gamma coefficient, *t*(55) = 1.50, *p* = .139, *d* = 0.20. The mixed-effects model analysis corroborated the conclusion that JOLs accurately predicted scene memorability. We used a logistic regression model with random slopes and intercepts for participants that predicted recognition hits from group-mean centered JOLs. This model showed that JOLs significantly predicted memory performance, *b* = 0.02, *z* = 9.03, *p* < .001, and it provided a significantly better fit to the data than an otherwise identical model without JOLs as a fixed-effects predictor, χ^2^(1) = 53.28, *p* < .001.

We also examined relative accuracy separately for each within-subject condition. As can be seen in Appendix Table 5, Gamma coefficients ranged between .19 and .35, were all significantly positive, and did not differ across within-subject conditions. In a mixed-effects model analysis, accuracy differences across conditions should produce significant interactions between the fixed-effects predictors for JOLs and for the manipulated cues. In a logistic regression model with random participant intercepts and random participant slopes for JOLs, we predicted recognition hits from group-mean centered JOLs, distinctiveness (0 = low, 1 = high), color (0 = grayscale, 1 = color), and their interactions. Results showed that neither distinctiveness nor color interacted with JOLs, Distinctiveness × JOLs: *b* = 0.001, *z* = 0.26, *p* = .798; Color × JOLs: *b* = 0.003, *z* = 1.14, *p* = .253; Distinctiveness × Color × JOLs: *b* = −0.010, *z* = 1.36, *p* = .175. Also, this model did not provide a better fit to the data than an otherwise identical model without interactions between JOLs and one or both cues, χ^2^(3) = 3.28, *p* = .350. Thus, the mixed-effects model analysis also demonstrated similar relative accuracy of JOLs across conditions.

### Discussion

As in previous work, contextual distinctiveness and color helped recognition memory. A new finding was that people based their JOLs for pictures of naturalistic scenes on contextual distinctiveness and color, with most but not all participants predicting better memory performance for distinct scenes and color scenes than for indistinct scenes and grayscale scenes. Individual-level analyses showed that many participants integrated both cues in their JOLs. Three different measures of relative accuracy converged on the conclusion that JOLs captured differences in the relative memorability of scenes and that relative accuracy was in the range reported in studies with verbal materials (e.g., Koriat, 2007; Rhodes, 2016; Bröder & Undorf, 2019). Overall, Experiment 1 revealed that the basis and accuracy of JOLs for naturalistic scenes was similar to that of JOLs for verbal materials.

## Experiment 2

Experiment 2 aimed to replicate and extend Experiment 1 by manipulating two intrinsic cues. As we mentioned in the Introduction, Isola et al. (2011) obtained independent ratings for various scene attributes from a norming sample and then assessed whether these ratings were predictive of picture memorability in a continuous recognition task completed by a new sample. Two of the picture attributes that Isola et al. (2011) found to predict memorability were the extent to which scenes tell a story and to which scenes are peaceful. Scenes that were judged to tell a story were remembered better than scenes that did not tell a story. Judged peacefulness, in contrast, was inversely related with scene memorability. In Experiment 2, participants studied, judged, and recognized scenes that were either low or high in story and peacefulness. We expected to replicate Isola et al.’s (2011) findings of better recognition memory for scenes high in story and worse recognition memory for scenes high in peacefulness. We expected that, as in Experiment 1, JOLs would be moderately predictive of recognition memory. It was unclear, however, whether story and peacefulness would affect JOLs and hence whether JOLs would accurately predict cue effects on recognition memory.

### Method

#### Participants

As in Experiment 1, we aimed at a sample size of 54 participants to obtain a statistical power of (1 − β) = .95 to detect medium-sized main effects (*f* = .25) with α = .05 when assuming a correlation of .50 between repeated measures. Participants were 54 University of Mannheim undergraduates (40 females, 14 males) with a mean age of 23.63 years (*SD* = 4.55).

#### Design and materials

**Fig. 3:**
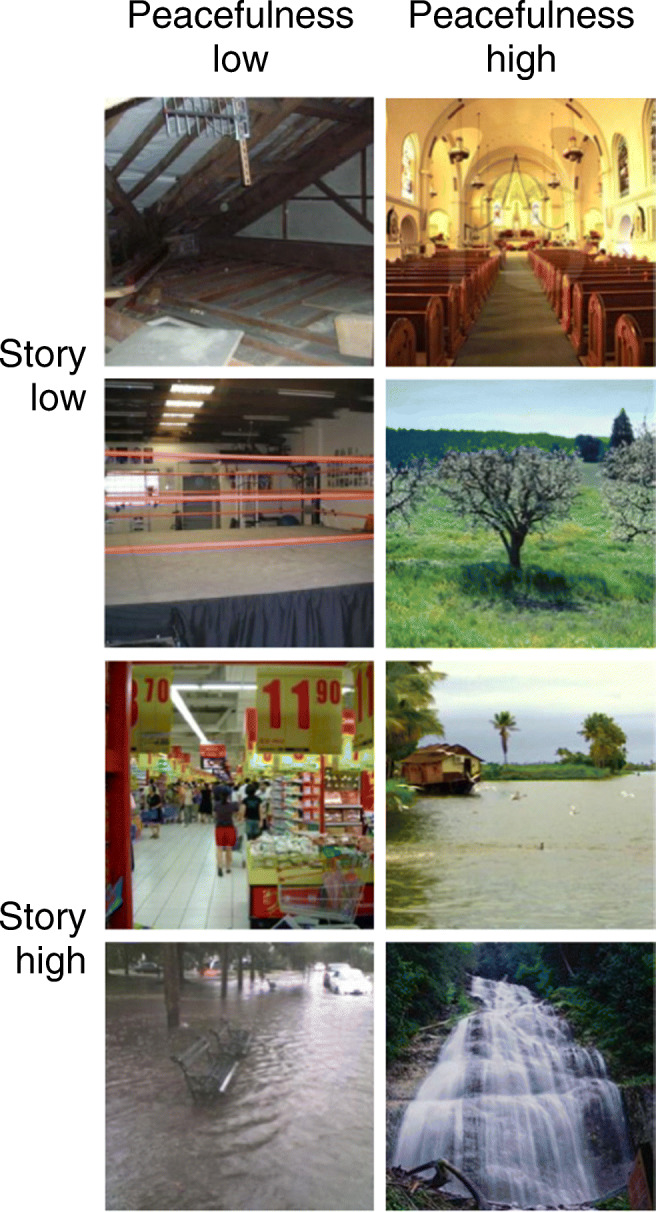
Example pictures used in Experiment 2

The design was a 2 (peacefulness: low vs. high) × 2 (story: low vs. high) within-participants design. Both factors were manipulated by selecting sets of normed scene pictures. Stimuli (see Fig. 3) were 240 pictures from the FIGRIM database (Bylinskii et al., 2015, mean ratings on story and peacefulness reported hereafter). Sixty scenes each were low in story and peacefulness (0.14 vs. 0.14), low in story and high in peacefulness (0.14 vs. 0.56), high in story and low in peacefulness (0.90 vs. 0.13), and high in story and peacefulness (0.89 vs. 0.55).^3^ Scenes from each combination of story and peacefulness were divided into two parallel sets with 30 pictures. For each participant, one set of scenes per combination of story and peacefulness served as targets and the second served as distractors. Across participants, picture sets were counterbalanced such that each picture served equally often as target and distractor. Eight additional scenes (two from each combination of story and peacefulness) served as primacy buffers to minimize primacy effects. Four of these scenes (one from each combination of story and peacefulness) were presented at the beginning of the study phase. All eight buffer scenes were presented at the beginning of the test phase. Primacy buffers were not analyzed.

#### Procedure

The procedure was identical to Experiment 1, except that all scenes were presented in color, that the study list included 120 regular scenes and four primacy buffers (resulting in 124 study trials), and that the test list included the 240 regular scenes and eight primacy buffers (resulting in 248 test trials).^4^

### Results

Story and peacefulness affected JOLs and recognition memory (see Table 1). A 2 (story: low vs. high) × 2 (peacefulness: low vs. high) repeated-measures ANOVA on JOLs revealed a main effect of story, *F*(1, 53) = 46.31, *p* < .001, η_p_^2^ = 0.47, indicating higher JOLs for scenes high rather than low in story and a significant interaction, *F*(1, 53) = 11.33, *p* = .001, η_p_^2^ = 0.18, but no main effect of peacefulness, *F* < 1. Follow-up *t* tests revealed no effect of peacefulness for scenes low in story, *t* < 1, but, for scenes high in story, marginally lower JOLs when peacefulness was high rather than low, *t*(53) = 1.98, *p* = .053, *d* = 0.27. A similar ANOVA on corrected hit rates *P*_r_ revealed main effects of story, *F*(1, 53) = 151.72, *p* < .001, η_p_^2^ = 0.74, and peacefulness, *F*(1, 53) = 28.66, *p* < .001, η_p_^2^ = 0.35, indicating that story helped memory but that peacefulness harmed memory. The interaction was not significant, *F*(1, 53) = 2.97, *p* = .091, η_p_^2^ = 0.05.

An individual-level analysis based on simple mean differences indicated that 46% of participants integrated story and peacefulness in their JOLs (see Table 2). An individual-level analysis based on Cohen’s *d* showed that most participants predicted better memory for scenes high in story, whereas approximately equal numbers of participants predicted that high peacefulness would help or harm memory (see Fig. 2). When using |*d*| ≥ .2 as a criterion, 41% of participants integrated story and peacefulness in their JOLs.

The gamma coefficient was significantly positive, *M* = .37 (*SD* = .21), *t*(53) = 13.10, *p* < .001, *d* = 1.80. The matching index *G* was also significantly positive, *M* = .24 (*SD* = .09), *t*(53) = 2.66, *p* = .010, *d* = 0.37, and numerically but not significantly lower than the gamma coefficient, *t*(53) = 1.37, *p* = .176, *d* = 0.19. A mixed-effects model analysis showed that JOLs significantly predicted memory performance, *b* = 0.04, *z* = 14.96, *p* < .001, and that including JOLs as a fixed-effects predictor significantly improved the fit of the model, χ^2^(1) = 91.78, *p* < .001. Thus, all three measures of resolution indicated that JOLs accurately predicted differences in the relative memorability of scenes.

Separate gamma coefficients for each within-subject condition (see Appendix Table 5) were higher for scenes high in story and low in peacefulness (.50) than for scenes low in story and high in peacefulness (.27). Neither of these coefficients differed from those in the other two conditions. All gamma coefficients were significantly positive. A mixed-effects model analysis revealed no significant interactions between the fixed-effects predictor JOLs and story or peacefulness, Story × JOLs: *b* = 0.006, *z* = 1.45, *p* = .148; Peacefulness × JOLs: *b* = −0.005, *z* = 1.49, *p* = .136; Story × Peacefulness × JOLs: *b* < −0.001, *z* = 0.08, *p* = .934. However, this model provided a significantly better fit to the data than an otherwise identical model without interactions between JOLs and one or both cues, χ^2^(3) = 8.97, *p* = .030. Overall, despite the significant likelihood-ratio test, gamma coefficients and regression coefficients showed little indication for differences in the relative accuracy of JOLs across conditions.

### Discussion

As in Isola et al. (2011), participants remembered scenes that were judged to tell a story better than scenes that did not tell a story, whereas they remembered peaceful scenes worse than scenes that were judged as not peaceful. At the aggregate level, people’s JOLs accurately predicted that story boosted memorability but failed to predict that peacefulness harmed memory. Individual-level analyses showed that nearly half of the participants integrated story and peacefulness into their JOLs. Also, individual-level analyses demonstrated that most participants predicted better memory for scenes high in story, whereas similar numbers of participants predicted that high peacefulness would help or harm memory. JOLs revealed robust relative accuracy.

Together, these results replicate Experiment 1 in showing that JOLs were moderately accurate and based on multiple cues. Moreover, the finding that cues can dissociate metamemory and actual memory—as was the case for peacefulness—is well known in the literature on JOLs for verbal materials (e.g., Dunlosky & Metcalfe, 2009; Koriat, 1997).

## Experiment 3

Experiment 3 aimed to replicate and extend the previous experiments to a situation where pictures of naturalistic scenes differed in five cues, two of which were extrinsic and three of which were intrinsic. Extrinsic cues were color and presentation frequency. Intrinsic cues were story, peacefulness, and whether scenes contained or did not contain persons. We predicted that effects of color, story, and peacefulness on recognition memory and JOLs would be similar to the previous experiments. In addition, we predicted better memory and higher JOLs for twice-presented than for once-presented scenes (e.g., Undorf et al., 2018) and better memory for scenes with than without persons (Isola et al., 2011). Whether the presence of persons would affect JOLs was an open question. Finally, we expected to replicate that JOLs are based on multiple cues and moderately accurate.

### Method

#### Participants

As in the previous experiments, we aimed at a sample size of 54 participants to obtain a statistical power of (1 − β) = .95 to detect medium-sized main effects and interactions (*f* = .25) with α = .05 when assuming a correlation of .50 between repeated measures. Participants were 62 University of Mannheim undergraduates, 10 of which did not take the delayed online memory test (see below). This left us with a final sample of *N* = 52 (46 females, six males, mean age of 20.52 years, *SD* = 3.52) and resulted in a slightly reduced actual statistical power of .9425 to detect *f* = .25 with the parameters used in the a priori power analysis.^5^

#### Design and materials

**Fig. 4:**
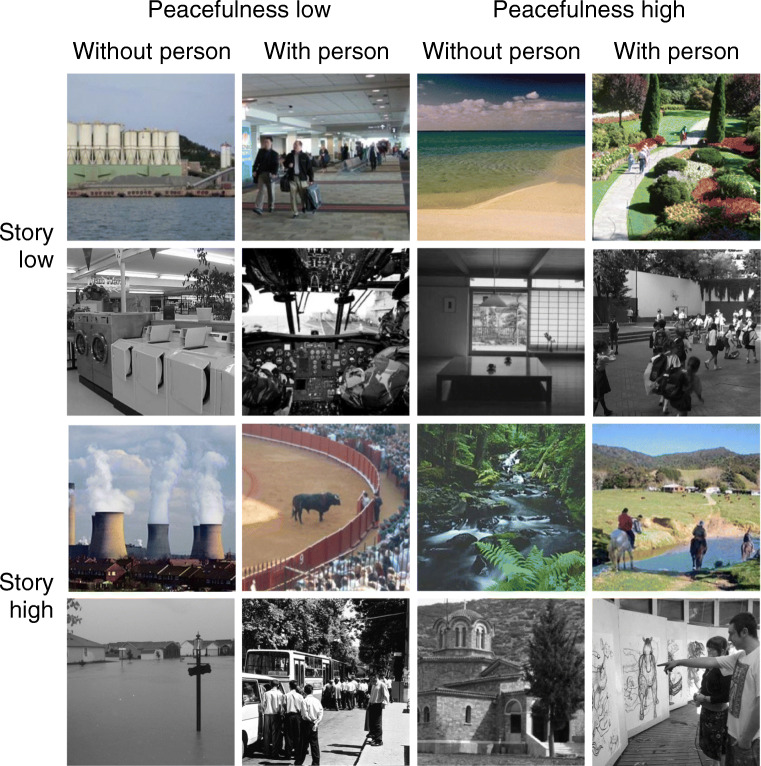
Example pictures used in Experiment 3

The design consisted of five within-participant factors: We selected eight sets of pictures representing all possible combinations of low and high peacefulness, low and high story, and scenes without and with persons. Stimuli (see Fig. 4) were 192 pictures from the FIGRIM database (Bylinskii et al., 2015, mean ratings reported hereafter). Twenty-four scenes each were low in story and peacefulness and without (0.25 vs. 0.19 vs. 0) or with (0.26 vs. 0.19 vs. 1) persons, low in story and high in peacefulness without (0.24 vs. 0.75 vs. 0) or with (0.24 vs. 0.74 vs. 1) persons, high in story and low in peacefulness without (0.58 vs. 0.20 vs. 0) or with (0.58 vs. 0.20 vs. 1) persons, and high in story and peacefulness without (0.58 vs. 0.75 vs. 0) or with (0.58 vs. 0.75 vs. 1) persons. Pictures from each combination of story, peacefulness, and person were divided into two parallel sets with 12 items each. For each participant, one set of scenes per cue combination served as targets and the second served as distractors. Across participants, picture sets were counterbalanced such that each picture served equally often as target and distractor.

#### Procedure

The procedure was identical to Experiment 1, with the following exceptions. Participants studied 96 scenes, with three randomly determined scenes from each combination of story, peacefulness, and person presented once in grayscale, twice in grayscale, once in color, and twice in color. To prevent ceiling effects in recognition memory performance due to repeated presentations, participants took the recognition test on the day after the study phase via the Internet. The mean delay between the beginning of the study phase and the beginning of the test phase was 24.79 hours (*SD* = 4.61).

### Results

**Fig. 5:**
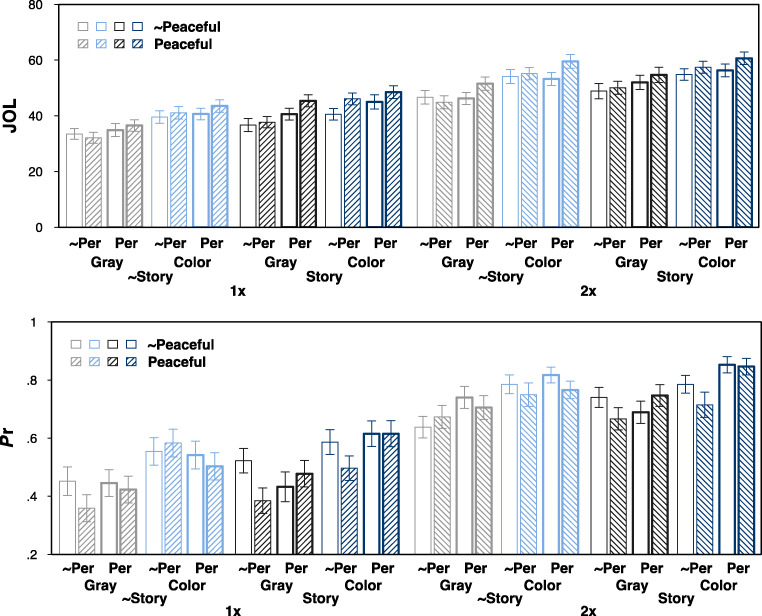
Mean judgments of learning (JOL; top panel) and corrected hit rates (*P*r; bottom panel) in Experiment 3. Error bars represent one standard error of the mean. ~Story = scenes low in story; Story = scenes high in story; ~Per = scenes without persons; Per = scenes with persons, ~Peaceful = scenes low in peacefulness; Peaceful = scenes high in peacefulness

For scenes that were studied twice, reported analyses are based only on JOLs from the second presentation (see Appendix 3 for JOLs from Presentation 1). Descriptive statistics for JOLs and recognition memory are presented in Fig. 5 and in Appendix 4. A 2 (presentation frequency) × 2 (color) × 2 (story) × 2 (peacefulness) × 2 (person) repeated-measures ANOVA on JOLs revealed main effects of all factors, indicating higher JOLs for twice-presented scenes, *F*(1, 51) = 94.88, *p* < .001, η_p_^2^ = 0.65, for colored scenes, *F*(1, 51) = 46.81, *p* < .001, η_p_^2^ = 0.48, for scenes that tell a story, *F*(1, 51) = 73.57, *p* < .001, η_p_^2^ = 0.59, for peaceful scenes, *F*(1, 51) = 25.80, *p* < .001, η_p_^2^ = 0.34, and for scenes with persons, *F*(1, 51) = 21.61, *p* < .001, η_p_^2^ = 0.30. The only significant interaction was between peacefulness and person, *F*(1, 51) = 6.43, *p* = .014, η_p_^2^ = 0.11, for all other interactions, *F ≤* 3.12, *p* ≥ .083. Separate ANOVAs for scenes with and without persons revealed that peacefulness increased JOLs only for scenes with persons, without persons: *F*(1, 51) = 2.72, *p* = .105, η_p_^2^ = 0.05, with persons: *F*(1, 51) = 29.71, *p* < .001, η_p_^2^ = 0.37.

A similar ANOVA on corrected hit rates *P*_r_ revealed four significant main effects, indicating better memory for twice-presented scenes, *F*(1, 51) = 278.87, *p* < .001, η_p_^2^ = 0.85, for colored scenes, *F*(1, 51) = 67.42, *p* < .001, η_p_^2^ = 0.57, for scenes with persons, *F*(1, 51) = 5.80, *p* = .020, η_p_^2^ = 0.10, and worse memory for peaceful scenes, *F*(1, 51) = 4.28, *p* = .044, η_p_^2^ = 0.08. A marginal main effect of story hinted that memory tended to be better for scenes that tell a story, *F*(1, 51) = 3.35, *p* = .073, η_p_^2^ = 0.06. Significant interactions were found between color, story, and person, *F*(1, 51) = 6.74, *p* = .012, η_p_^2^ = 0.12, and between story, peacefulness, and person, *F*(1, 51) = 9.70, *p* = .003, η_p_^2^ = 0.16. No other interactions were significant, *F ≤* 2.76, *p* ≥ .103.

Two complementary individual-level analyses (see Table 2) revealed that more than 94% of participants integrated at least two cues in their JOLs, with 19% (simple mean differences) or 35% of participants (|*d*| ≥ .2) even integrating four or five cues. We also examined how many participants based their JOLs on the two cues manipulated in Experiment 2. The percentages of participants who integrated the two cues story and peacefulness in their JOLs amounted to 9.62% (five participants) according to simple mean differences and to 19.23% (10 participants) when using |*d*| ≥ .2 as a criterion. This shows that although many more participants from Experiment 3 than from Experiment 2 based their JOLs on multiple cues, fewer participants from the former than from the latter experiment based their JOLs on both story and peacefulness.

The gamma coefficient was significantly positive, *M* = .45 (*SD* = .19), *t*(51) = 17.00, *p* < .001, *d* = 2.38. The matching index *G* was also significantly positive, *M* = .59 (*SD* = .06), *t*(51) = 10.30, *p* < .001, *d* = 1.44, and higher than the gamma coefficient, *t*(51) = 3.01, *p* = .004, *d* = 0.42. A mixed-effects model analysis showed that JOLs significantly predicted memory performance, *b* = 0.04, *z* = 13.93, *p* < .001, and that including JOLs as a fixed-effects predictor significantly improved the fit of the model, χ^2^(1) = 85.92, *p* < .001. Thus, all three measures of relative accuracy indicated that JOLs accurately predicted differences in the relative memorability of scenes.

Separate gamma coefficients for each within-subject condition (see Appendix Table 6) ranged from −.10 to .33 and did not differ across conditions. Approximately half of the coefficients were significantly positive and the remaining coefficients did not differ from zero. A mixed-effects model revealed that none of the manipulated cues or combination of manipulated cues interacted with JOLs, |*b*| ≤ 0.052, *z* ≤ 1.78, *p* ≥ .075. This model did not provide a better fit to the data than an otherwise identical model without interactions between JOLs and the manipulated cues, χ^2^(31) = 32.82, *p* = .378. Thus, gamma coefficients and mixed-effects models likewise suggested that relative accuracy of JOLs was similar across conditions.

### Discussion

All five cues had the expected effects on recognition memory: Two presentations, color, story, and person improved memory, whereas peacefulness reduced memory. For once-presented scenes, the 24-hour retention interval resulted in similar recognition memory as in Experiment 1, but in worse recognition memory than in Experiment 2. 

At the aggregate level, people’s JOLs accurately predicted the beneficial effects of presentation frequency, color, story, and person on memory, but again failed to predict that peacefulness harmed memory. Individual-level analyses showed that above 96% of participants integrated two or more cues into their JOLs. At the same time, the proportion of participants who based their JOLs on the cues story and peacefulness was lower than in Experiment 2, in which only these two cues were manipulated. This finding might be related to reduced saliency of individual cues due to the simultaneous manipulation of five cues: If cue use in JOLs is a strategic process—as has been suggested empirically by Undorf and Bröder (2020) for verbal materials—participants may select a couple of cues from those available at study to base their JOLs on. If so, in Experiment 3, they did not necessarily select the subset of cues that we manipulated in Experiment 2. 

As in Experiments 1 and 2, JOLs revealed robust relative accuracy. Overall, Experiment 3 fully replicated the previous experiments in demonstrating that JOLs were based on multiple cues and moderately accurate.

## Cue integration and memory performance

The data obtained in this study allowed us examine the additional question whether cue integration in JOLs plays a functional role in enhancing memory performance.^6^ To address this question, we compared recognition performance across participants who differed in cue integration. Thus, we submitted corrected hit rates *P*_r_ from each experiment to mixed ANOVAs that included one measure of cue integration at the individual level as a between-subjects factor (simple mean differences or Cohen’s *d*) and the manipulated cues as repeated-measures factors. In Experiments 1 and 2, we compared participants who based their JOLs on not more than one manipulated cue with those who based their JOLs on both manipulated cues. In Experiment 3, where nearly all participants based their JOLs on at least two cues, we compared participants who based their JOLs on one or two manipulated cues with those who based their JOLs on three or more manipulated cues. For reasons of conciseness, we report only main effects of cue integration and interactions involving cue integration.

When defining cue integration as simple mean differences, Experiment 1 revealed a main effect of cue integration, *F*(1, 54) = 6.63, *p* = .013, η_p_^2^ = 0.11, indicating better recognition performance in participants who integrated contextual distinctiveness and color in their JOLs, and a significant interaction between cue integration and distinctiveness, *F*(1, 54) = 6.63, *p* = .013, η_p_^2^ = 0.11. Follow-up *t* tests revealed a stronger effect of distinctiveness on recognition performance in participants who integrated both cues than in participants who did not integrate the two cues, *t*(36) = 7.90, *p* < .001, *d* = 1.32 vs. *t*(18) = 2.81, *p* = .012, *d* = 0.66. Neither the interaction of cue integration and color nor the three-way interaction were significant, *F* < 1. In Experiment 2, there was no main effect of cue integration and none of the interactions involving cue integration were significant, *F* < 1. In Experiment 3, there was a significant interaction among high levels of cue integration, presentation frequency, story, and peacefulness, *F*(1, 50) = 5.70, *p* = .021, η_p_^2^ = 0.10. Separate ANOVAs for participants with low and high levels of cue integration revealed that the Presentation Frequency × Story × Peacefulness interaction was more pronounced in participants with low levels of cue integration. Neither the main effect of levels of cue integration nor any other interaction involving levels of cue integration were significant, *F* ≤ 3.41, *p* ≥ .071.

When defining cue integration as mean differences of |Cohen’s *d*| ≥ .2, neither Experiment 1 nor Experiment 2 showed a main effect of cue integration or any significant interactions involving cue integration, Experiment 1: *F* ≤ 2.48, *p* ≥ .121; Experiment 2: *F* < 1. In Experiment 3, there was a significant interaction among high levels of cue integration, presentation frequency, and person, *F*(1, 50) = 4.55, *p* = .038, η_p_^2^ = 0.08. Separate ANOVAs for participants with low and high levels of cue integration revealed a significant interaction between presentation frequency and person in participants with low levels of cue integration, *F*(1, 16) = 6.68, *p* = .020, η_p_^2^ = 0.30, but not in participants with high levels of cue integration, *F* < 1. Neither the main effect of levels of cue integration nor any other interactions involving levels of cue integration were significant, *F* ≤ 3.24, *p* ≥ .077.

Overall, there was little evidence for a relationship between cue integration in JOLs and recognition performance. Cue integration defined in terms of simple mean differences was related to recognition performance in Experiment 1 but not in Experiments 2 and 3. When defining cue integration in terms of effect sizes, none of the three experiments showed a relationship between cue integration and good recognition performance. As we will consider in the General Discussion, it is nevertheless possible that, at least for some cues, cue integration is related to good memory performance.

## General Discussion

This research provided important new insights into the basis and accuracy of metamemory for pictures of naturalistic scenes. First, JOLs for scene pictures were based on multiple probabilistic cues. Specifically, we identified three intrinsic cues (tells a story, peaceful, contains persons) and three extrinsic cues (color, contextual distinctiveness, presentation frequency) that underlay JOLs. In each experiment, all manipulated cues affected JOLs at the aggregate level. Individual-level analyses revealed that between 41% (Experiment 2) and 96% (Experiment 3) of participants integrated two or more cues in their JOLs. Notably, these numbers are probably lower-bound estimates of cue integration. Since our analyses focused solely on experimentally *manipulated* cues, it would go unnoticed if participants based their JOLs on other scene attributes. For example, it is unlikely that the small percentage of participants classified as using zero cues in Table 2 did not base their JOLs on any cues. Rather, it is likely that they merely based their JOLs on none of the experimentally manipulated cues.

Second, three measures of relative accuracy—the within-subject gamma correlation between JOLs and memory performance, the matching index *G* from Brunswik’s (1952) lens model (Bröder & Undorf, 2019), and a mixed-effects model analysis (Murayama et al., 2014)—all showed that JOLs were moderately accurate. This convergence demonstrates that notwithstanding several shortcomings (e.g., Benjamin & Diaz, 2008; Masson & Rotello, 2009; Spellman et al., 2008), the gamma coefficient provided valid conclusions about the relative accuracy of JOLs.

Finally, examining relative accuracy for each cue combination separately using the gamma coefficient and mixed-effects models revealed above-chance accuracy that did not differ across within-subjects conditions in Experiments 1 and 2. These findings suggest that JOLs accurately predicted relative differences in memorability across scenes that were similar regarding the two manipulated cues. Experiment 3 showed a somewhat different pattern. As in the previous experiments, gamma coefficients and mixed-effects models indicated similar relative accuracy across cue combinations. At the same time, gamma correlations did not differ reliably from zero for approximately half of the cue combinations. It might be possible that this reflects people’s inability to predict differences in memorability across sets of scenes that are similar regarding the five cues manipulated in Experiment 3. More likely, however, is that insignificant gamma coefficients were due to the very small number of items per cue combination. Consistent with this idea, the mixed-effects model analysis showed that JOLs accurately predicted recognition performance in Experiment 3. In any case, the present study showed that JOLs were similar in relative accuracy across within-subjects conditions. From a methodological perspective, it demonstrated that analyzing JOL accuracy using mixed-effects models offers a methodological advantage over the gamma coefficient when separately analyzing multiple within-subjects conditions each of which includes only a limited number of trials.

We simultaneously manipulated two or more picture attributes that previous studies found to affect recognition memory for pictures of naturalistic scenes (Bylinskii et al., 2015; Isola et al., 2011, 2014). Our results fully replicated these prior studies. Specifically, we obtained better recognition memory for scenes independent raters judged to tell a story than for scenes that did not tell a story, better memory for contextually distinct than indistinct scenes, better memory for scenes with than without persons, and worse memory for peaceful scenes than for scenes that were not peaceful. This replication was not trivial, given a number of procedural differences across experiments. We used old–new recognition tasks, whereas Bylinskii et al. (2015) and Isola et al. (2011, 2014) used continuous recognition tasks. Unlike Bylinskii et al. (2015) and Isola et al. (2011, 2014), we obtained item-by-item JOLs at study. Also, our participants were undergraduates, whereas participants in Bylinskii et al. (2015) and Isola et al.’s (2011, 2014) studies came from the general population.

Our findings on the basis and accuracy of JOLs are perfectly consistent with the few previous studies on JOLs for naturalistic pictures (Hourihan & Bursey, 2017; Kao et al., 2005; Schmoeger et al., 2020; Tauber et al., 2017). More importantly, they demonstrate close parallels between metamemory for naturalistic pictures and verbal materials. In both domains, JOLs are based on multiple intrinsic and extrinsic cues (for evidence from studies using verbal materials, see Bröder & Undorf, 2019; Tatz & Peynircioğlu, 2019; Bröder & Undorf, 2019; Undorf et al., 2018). While most cues have similar effects on JOLs and memory performance, some cue effects are not captured by JOLs. Besides showing that metamemory is fallible, this demonstrates that metamemory judgments are based on the manipulated cues rather than on direct access to an internal memory representation (e.g., Koriat, 1997, 2007; Undorf, 2020). Also, the moderate levels of relative JOL accuracy found in the current research correspond well with the findings from studies with verbal materials (e.g., Dunlosky & Metcalfe, 2009; Koriat, 2007; Rhodes, 2016). Overall, this demonstrates that established principles and findings in the literature on metamemory generalize to pictures of naturalistic scenes.

It is instructive to note that the JOLs obtained in this study were much more accurate than the memorability ratings obtained by Isola et al. (2011, 2014). Isola et al. found that binary responses to the question whether each of a set of scenes was or was not memorable or whether people would or would not, when seeing each scene again later, realize having seen it before were not at all predictive of scene memorability. We can only speculate about the reasons for these differences in findings. One possibility is that Isola et al. (2014) underestimated people’s metacognitive ability because they solicited binary judgments for a small sample of pictures outside of a learning phase. In contrast, in our experiments, people completed a study phase with fine-grained item-by-item JOLs on a large set of scenes that varied on various picture attributes and/or extrinsic cues. In line with this speculation, Hourihan and Bursey (2017) and Tauber et al. (2017) found reliable effects of valence on older and younger adults’ JOLs for naturalistic pictures when soliciting fine-grained JOLs for 90 or more pictures during a study phase, whereas Tomaszczyk and Fernandes (2013), who asked participants to select 15 memorable pictures out of a set of 30 pictures, found valence effects for older but not younger adults. Also, evidence suggests that ease-of-learning judgments made before studying materials have lower relative accuracy than JOLs (e.g., Jönsson & Kerimi, 2011; Kelemen et al., 2000; Leonesio & Nelson, 1990; Pieger et al., 2016; Watier & Collin, 2011, 2012). Another possibility is that accuracy differences between Isola et al.’s (2014) memorability judgments and JOLs from the current experiments were connected to the fact that Isola et al. (2014) related people’s memorability ratings to average recognition memory, whereas we related each person’s JOLs to his or her own recognition memory. Basing metamemory judgments on idiosyncratic person-item interactions (e.g., “I will remember this picture of an airplane because I collect airplane models”) presumably reduced relative accuracy of Isola et al.’s (2014) memorability ratings but increased relative accuracy of JOLs in the current experiments (see Bröder & Undorf, 2019; Bröder & Undorf, 2019).

Of course, many questions remain regarding the cues that underlie JOLs for naturalistic scene pictures. First, scene pictures possess a multitude of attributes. For instance, Isola et al. (2011) came up with more than 900 features that characterize scene pictures. It is obvious that many more cues than the ones we identified in this study could potentially affect people’s metamemory judgments. As it was impossible to control for all these potential cues, we cannot exclude the possibility that some picture attributes or attribute combinations are correlated with the cues we manipulated in the current study. At the same time, however, some cues or cue combinations that affect metamemory probably varied across scenes that were similar regarding manipulated cues. For instance, Experiment 3 revealed effects of whether scenes contained or did not contain persons even though the pictures with persons differed in whether faces were visible or not, a feature that boosts memorability according to Isola et al. (2011). This highlights that the cues we manipulated in the present study had robust effects on JOLs and recognition memory. 

Moreover, while we found little evidence for differences between intrinsic and extrinsic cues in cue use or cue integration, it is possible that important differences between cues exist. In the present study, integrating contextual distinctiveness in JOLs was related to good recognition performance, whereas integrating any other cue in JOLs was unrelated to actual memory. Distinctiveness differed from the other cues in that it required some experience with the study task and, presumably, became apparent only after a couple of study trials (see also Matvey et al., 2006). Maybe, sensitivity to changes in cue effects across study trials is associated with participant characteristics that boost memory performance. Promising candidates may include motivation and intellectual ability. Notably, targeting changes in cue effects across study trials would require modifications to the experimental procedures used in this study and many other JOL studies. For instance, it would be important to control the order of stimuli at study rather than using new a random order for each participant.

By showing that people have the remarkable ability to integrate up to five cues in their JOLs, the present study extends previous studies on cue integration in metamemory judgments. Hitherto, studies with verbal materials simultaneously manipulated a maximum of four cues (Undorf et al., 2018). Pictures of naturalistic scenes, which possess a variety of attributes, may be well suited to investigate possible limits of information integration in metamemory judgments. 

One limitation of the approach used here and in our previous research (Undorf & Bröder, 2020; Undorf et al., 2018) is that estimates of the number of cues integrated or the number of people integrating a certain number of cues are always restricted to the cues that are manipulated or at least measured. As it evades the experimenter’s knowledge whether and how many cues people use *in addition* to the manipulated cues, it may not be appropriate to take the resulting numbers too seriously. This reasoning may also explain why the number of participants apparently integrating at least two cues was much higher in Experiment 3 than in Experiments 1 and 2: Varying (and thus observing) more picture attributes increased our chances to include cues people used. Also, the ensuing uncertainty about the number of cues participants actually used for their JOLs might have obscured beneficial effects of cue integration on recognition performance. Despite this limitation, the current study clearly shows that people integrate multiple cues in their JOLs.

In conclusion, this study demonstrates that the exceptionally good memory for naturalistic pictures is complemented by moderately accurate metamemory stemming from reliance on multiple probabilistic cues. The current research thereby demonstrates close parallels between metamemory for naturalistic pictures and verbal materials, indicating that established principles and findings in metamemory research generalize to new materials.

## Appendix 1: Number of stimuli in each within-subject condition

Appendix Tables 3 and 4 present the number of stimuli in each within-subject condition of Experiments 1 and 2, and of Experiment 3, respectively

**Table 3:** Number of stimuli in the recognition test for each within-subject condition of Experiments 1 and 2

		Number of targets	Number of distractors
Experiment 1			
Indistinct 1	Grayscale	14	14
	Color	14	14
Indistinct 2	Grayscale	14	14
	Color	14	14
Indistinct 3	Grayscale	14	14
	Color	14	14
Distinct 1	Grayscale	2	2
	Color	2	2
Distinct 2	Grayscale	2	2
	Color	2	2
Distinct 3	Grayscale	2	2
	Color	2	2
		Σ 96	Σ 96
Experiment 2			
Story low	Peacefulness low	30	30
	Peacefulness high	30	30
Story high	Peacefulness low	30	30
	Peacefulness high	30	30
		Σ 120	Σ 120

**Table 4:** Number of stimuli in the recognition test for each within-subject condition of Experiment 3

					Number of targets	Number of distractors
1×	~Story	Grayscale	~Person	~Peaceful	3	3
				Peaceful	3	3
			Person	~Peaceful	3	3
				Peaceful	3	3
		Color	~Person	~Peaceful	3	3
				Peaceful	3	3
			Person	~Peaceful	3	3
				Peaceful	3	3
	Story	Grayscale	~Person	~Peaceful	3	3
				Peaceful	3	3
			Person	~Peaceful	3	3
				Peaceful	3	3
		Color	~Person	~Peaceful	3	3
				Peaceful	3	3
			Person	~Peaceful	3	3
				Peaceful	3	3
2×	~Story	Grayscale	~Person	~Peaceful	3	3
				Peaceful	3	3
			Person	~Peaceful	3	3
				Peaceful	3	3
		Color	~Person	~Peaceful	3	3
				Peaceful	3	3
			Person	~Peaceful	3	3
				Peaceful	3	3
	Story	Grayscale	~Person	~Peaceful	3	3
				Peaceful	3	3
			Person	~Peaceful	3	3
				Peaceful	3	3
		Color	~Person	~Peaceful	3	3
				Peaceful	3	3
			Person	~Peaceful	3	3
				Peaceful	3	3
					Σ 96	Σ 96

## Appendix 2: Gamma Coefficients in Each Within-Subject condition

Appendix Tables 5 and 6 present the gamma correlation between JOLs and recognition hits in each within-subject condition of Experiments 1 and 2, and of Experiment 3, respectively.

**Table 5:** Mean (and standard deviation) of the gamma correlation between JOLs and recognition hits in each within-subject condition of Experiments 1 and 2

		Gamma	*t*	*df*	*p*	*d*
Experiment 1						
Indistinct	Grayscale	.20_a_ (.26)	5.79	55	<.001	0.78
	Color	.31_a_ (.29)	7.94	55	<.001	1.07
Distinct	Grayscale	.35_a_ (.57)	4.58	55	<.001	0.62
	Color	.19_a_ (.59)	2.38	55	.021	0.32
Experiment 2						
~Story	~Peaceful	.37_ab_ (.41)	6.57	53	<.001	0.90
	Peaceful	.27_a_ (.26)	7.67	53	<.001	1.05
Story	~Peaceful	.50_b_ (.30)	12.11	53	<.001	1.66
	Peaceful	.37_ab_ (.37)	7.22	53	<.001	0.99

Within each experiment, means with different subscripts are different at *p* < .05 according to Tukey’s HSD tests

**Table 6:** Mean (and standard deviation) of the gamma correlation between JOLs and recognition hits in each within-subject condition of Experiment 3

					Gamma	*t*	*df*	*p*	*d*
1×	~Story	Grayscale	~Person	~Peaceful	.15_a_ (.67)	1.66	51	.103	0.23
				Peaceful	.27_a_ (.66)	2.94	51	.005	0.41
			Person	~Peaceful	.13_a_ (.63)	1.55	51	.128	0.22
				Peaceful	.12_a_ (.76)	1.10	51	.278	0.15
		Color	~Person	~Peaceful	.12_a_ (.70)	1.18	51	.243	0.17
				Peaceful	.15_a_ (.64)	1.74	51	.088	0.24
			Person	~Peaceful	.06_a_ (.70)	0.60	51	.554	0.08
				Peaceful	.15_a_ (.57)	1.93	51	.059	0.27
	Story	Grayscale	~Person	~Peaceful	.29_a_ (.67)	3.12	51	.003	0.44
				Peaceful	.33_a_ (.71)	3.34	51	.002	0.47
			Person	~Peaceful	.19_a_ (.66)	2.11	51	.040	0.30
				Peaceful	.33_a_ (.71)	3.34	51	.002	0.47
		Color	~Person	~Peaceful	.15_a_ (.70)	1.59	51	.118	0.22
				Peaceful	.19_a_ (.69)	2.02	51	.049	0.28
			Person	~Peaceful	.23_a_ (.70)	2.37	51	.022	0.33
				Peaceful	.06_a_ (.61)	0.68	51	.497	0.10
2×	~Story	Grayscale	~Person	~Peaceful	.10_a_ (.63)	1.09	51	.280	0.15
				Peaceful	.14_a_ (.60)	1.63	51	.109	0.23
			Person	~Peaceful	−.10_a_ (.57)	1.22	51	.229	0.17
				Peaceful	.27_a_ (.53)	3.68	51	<.001	0.51
		Color	~Person	~Peaceful	.17_a_ (.43)	2.90	51	.005	0.41
				Peaceful	.25_a_ (.52)	3.47	51	.001	0.49
			Person	~Peaceful	.10_a_ (.52)	1.40	51	.168	0.20
				Peaceful	.14_a_ (.49)	2.00	51	.051	0.28
	Story	Grayscale	~Person	~Peaceful	.02_a_ (.58)	0.24	51	.811	0.03
				Peaceful	.10_a_ (.50)	1.40	51	.168	0.20
			Person	~Peaceful	.10_a_ (.57)	1.22	51	.229	0.17
				Peaceful	.13_a_ (.44)	2.19	51	.033	0.31
		Color	~Person	~Peaceful	.19_a_ (.53)	2.64	51	.011	0.37
				Peaceful	.21_a_ (.54)	2.84	51	.006	0.40
			Person	~Peaceful	.14_a_ (.40)	2.44	51	.018	0.34
				Peaceful	.17_a_ (.38)	3.27	51	.002	0.46

Means with different subscripts are different at *p* < .05 according to Tukey’s HSD tests

## Appendix 3: JOLs from the first study presentation of twice-presented scenes in Experiment 3

JOLs from the first study presentation of twice-presented scenes in Experiment 3 (see Table 1) were very similar to JOLs for once-studied scenes (see Appendix Table 7).

A 2 (color) × 2 (story) × 2 (peacefulness) × 2 (person) repeated-measures ANOVA revealed main effects for all four factors, indicating higher JOLs for colored scenes, *F*(1, 51) = 27.22, *p* < .001, η_p_^2^ = 0.35, for scenes that tell a story, *F*(1, 51) = 32.47, *p* < .001, η_p_^2^ = 0.39, for peaceful scenes, *F*(1, 51) = 10.57, *p* = .002, η_p_^2^ = 0.17, for scenes with persons, *F*(1, 51) = 5.18, *p* = .027, η_p_^2^ = 0.09, and significant interactions between peacefulness and person, *F*(1, 51) = 12.86, *p* < .001, η_p_^2^ = 0.20, and between story, peacefulness, and person, *F*(1, 51) = 4.12, *p* = .048, η_p_^2^ = 0.08. No other interactions were significant, *F ≤* 1.41, *p* ≥ .241.

**Table 7:** Means (and standard deviations) of judgments of learning (JOL) from the first study presentations of twice-presented scenes in Experiment 3

				JOL
~Story	Grayscale	~Person	~Peaceful	34.94 (16.47)
			Peaceful	30.39 (13.47)
		Person	~Peaceful	32.43 (14.24)
			Peaceful	40.83 (16.45)
	Color	~Person	~Peaceful	40.06 (17.50)
			Peaceful	40.77 (14.62)
		Person	~Peaceful	39.04 (15.47)
			Peaceful	46.03 (18.46)
Story	Grayscale	~Person	~Peaceful	39.30 (17.10)
			Peaceful	39.36 (15.80)
		Person	~Peaceful	38.59 (17.05)
			Peaceful	42.12 (18.67)
	Color	~Person	~Peaceful	43.65 (15.53)
			Peaceful	45.83 (15.88)
		Person	~Peaceful	43.97 (17.48)
			Peaceful	48.65 (14.76)

~*Story* scenes low in story, *Story* scenes high in story, ~*Person* scenes without persons; *Person* scenes with persons, ~*Peaceful* scenes low in peacefulness; *Peaceful* scenes high in peacefulness

## Appendix 4: Descriptive statistics for JOLs and recognition memory in Experiment 3

Appendix Table 8 presents descriptive statistics for JOLs and recognition memory for each within-subject condition in Experiment 3.

**Table 8:** Descriptive statistics for Experiment 3

					JOL	% Hits	% False alarms	*P*_r_
1×	~Story	Grayscale	~Person	~Peaceful	33.46 (13.92)	61.54 (31.92)	16.35 (18.22)	.45 (.35)
				Peaceful	32.12 (14.31)	50.00 (31.31)	14.10 (21.23)	.36 (.34)
			Person	~Peaceful	34.87 (16.63)	55.13 (32.93)	10.58 (16.84)	.45 (.33)
				Peaceful	36.54 (14.99)	52.56 (30.50)	10.26 (13.67)	.42 (.33)
		Color	~Person	~Peaceful	39.55 (15.99)	66.67 (31.66)	11.22 (16.07)	.55 (.34)
				Peaceful	41.09 (16.29)	64.74 (31.25)	6.41 (11.02)	.58 (.34)
			Person	~Peaceful	40.64 (14.66)	60.90 (32.15)	6.73 (11.56)	.54 (.34)
				Peaceful	43.53 (16.04)	61.54 (35.17)	11.22 (14.66)	.50 (.34)
	Story	Grayscale	~Person	~Peaceful	36.67 (16.75)	61.54 (29.80)	9.29 (15.27)	.52 (.30)
				Peaceful	37.69 (14.47)	54.49 (30.98)	16.03 (17.45)	.38 (.32)
			Person	~Peaceful	40.58 (15.25)	55.77 (34.75)	12.50 (14.34)	.43 (.37)
				Peaceful	45.39 (15.41)	59.62 (30.49)	11.86 (16.28)	.48 (.33)
		Color	~Person	~Peaceful	40.51 (14.97)	64.74 (29.82)	6.09 (9.35)	.59 (.31)
				Peaceful	46.09 (15.28)	58.33 (31.57)	8.65 (11.66)	.50 (.31)
			Person	~Peaceful	45.00 (18.64)	67.31 (30.60)	5.77 (13.56)	.62 (.32)
				Peaceful	48.53 (16.55)	69.23 (31.55)	7.69 (12.98)	.62 (.32)
2×	~Story	Grayscale	~Person	~Peaceful	46.60 (17.56)	80.13 (21.14)	16.35 (18.22)	.64 (.27)
				Peaceful	44.87 (16.96)	81.41 (23.26)	14.10 (21.23)	.67 (.29)
			Person	~Peaceful	46.22 (15.54)	84.62 (22.35)	10.58 (16.84)	.74 (.28)
				Peaceful	51.54 (17.07)	80.77 (27.49)	10.26 (13.67)	.71 (.29)
		Color	~Person	~Peaceful	54.10 (18.16)	89.74 (19.29)	11.22 (16.07)	.79 (.23)
				Peaceful	55.13 (15.61)	81.41 (26.74)	6.41 (11.02)	.75 (.29)
			Person	~Peaceful	53.21 (16.93)	88.46 (18.54)	6.73 (11.56)	.82 (.20)
				Peaceful	59.55 (17.63)	87.82 (20.90)	11.22 (14.66)	.77 (.21)
	Story	Grayscale	~Person	~Peaceful	48.85 (19.73)	83.33 (22.39)	9.29 (15.27)	.74 (.25)
				Peaceful	50.06 (16.95)	82.69 (25.98)	16.03 (17.45)	.67 (.28)
			Person	~Peaceful	51.99 (18.39)	81.41 (23.26)	12.50 (14.34)	.69 (.27)
				Peaceful	54.68 (19.95)	86.54 (22.15)	11.86 (16.28)	.75 (.27)
		Color	~Person	~Peaceful	54.81 (15.12)	84.62 (19.20)	6.09 (9.35)	.79 (.22)
				Peaceful	57.44 (15.60)	80.13 (26.62)	8.65 (11.66)	.71 (.31)
			Person	~Peaceful	56.28 (16.33)	91.03 (17.61)	5.77 (13.56)	.85 (.20)
				Peaceful	60.64 (16.02)	92.31 (15.64)	7.69 (12.98)	.85 (.21)

~*Story* scenes low in story; *Story* scenes high in story; ~*Person* scenes without persons; *Person* scenes with persons, ~*Peaceful* scenes low in peacefulness; *Peaceful* scenes high in peacefulness

## Footnotes

1 Notably, JOLs for verbal materials have been found to be highly accurate when solicited after a delay instead of immediately after study (e.g., Nelson & Dunlosky, 1991; Rhodes & Tauber, 2011; Undorf et al., 2016). The present study, however, focused on immediate JOLs.

2 The classification of color as an extrinsic cue follows from our definition of extrinsic cues as cues that can be randomly assigned to study items as opposed to intrinsic cues that are inseparable from study items. Alternatively, color might be considered an intrinsic cue, because it concerns properties inherent to the picture (for a similar discussion, see Undorf et al., 2018). Note, however, that the classification of color or any other cue as extrinsic or intrinsic does not affect the current theoretical aims and conclusions.

3 In response to a reviewer comment, we tested for correlations between the cues manipulated in Experiments 1 and 2 and the cues manipulated in Experiment 3. Results indicated that whether or not scenes contained persons was moderately correlated with story (*r* = .44) and peacefulness (*r* = −.31). This confound was removed in Experiment 3 where we also found significant effects of story and peacefulness on JOLs.

4 We did not use primacy buffers in Experiment 1 so as not to interfere with the contextual distinctiveness manipulation that required scenes to come from a small set of categories with identical numbers of scenes per distinct and indistinct category. Similarly, the need to counterbalance five cues prevented us from using primacy buffers in Experiment 3.

5 All JOL results were unchanged when including the full sample.

6 We thank the action editor for suggesting this interesting perspective.
